# Association of Hospital Public Quality Reporting With Electronic Health Record Medication Safety Performance

**DOI:** 10.1001/jamanetworkopen.2021.25173

**Published:** 2021-09-21

**Authors:** A. Jay Holmgren, David W. Bates

**Affiliations:** 1 University of California, San Francisco, San Francisco; 2 Brigham & Women’s Hospital, Harvard Medical School, Harvard T.H. Chan School of Public Health, Boston, Massachusetts

## Abstract

**Importance:**

Despite billions spent in public investment, electronic health records (EHRs) have not delivered on the promise of large quality and safety improvement. Simultaneously, there is debate on whether public quality reporting is a useful tool to incentivize quality improvement.

**Objective:**

To evaluate whether publicly reported feedback was associated with hospital improvement in an evaluation of medication-related clinical decision support (CDS) safety performance.

**Design, Settings, and Participants:**

This nonrandomized controlled trial included US hospitals that participated in the Computerized Provider Order Entry (CPOE) Evaluation Tool in the Leapfrog Hospital Survey, a national quality reporting program that evaluates safety performance of hospital CDS using simulated orders and patients, in 2017 to 2018. A sharp regression discontinuity design was used to identify the association of receiving negative feedback with hospital performance improvement in the subsequent year. Data were analyzed from January through September 2020.

**Exposures:**

Publicly reported quality feedback.

**Main Outcomes and Measures:**

The main outcome was improvement from 2017 to 2018 on the Leapfrog CPOE Evaluation Tool, using regression discontinuity model estimates of the association of receiving negative publicly reported feedback with quality improvement.

**Results:**

A total of 1183 hospitals were included, with a mean (SD) CPOE score of 59.3% (16.3%) at baseline. Hospitals receiving negative feedback improved 8.44 (95% CI, 0.09 to 16.80) percentage points more in the subsequent year compared with hospitals that received positive feedback on the same evaluation. This change was driven by differences in improvement in basic CDS capabilities (β = 8.71 [95%CI, 1.67 to 18.73]) rather than advanced CDS (β = 6.15 [95% CI, −9.11 to 26.83]).

**Conclusions and Relevance:**

In this nonrandomized controlled trial, publicly reported feedback was associated with quality improvement, suggesting targeted measurement and reporting of process quality may be an effective policy lever to encourage improvement in specific areas. Clinical decision support represents an important tool in ensuring patient safety and decreasing adverse drug events, especially for complex patients and those with multiple chronic conditions who often receive several different drugs during an episode of care.

## Key Points

**Question:**

Is receiving publicly reported negative feedback regarding EHR medication safety associated with hospitals improving their performance in the next year?

**Findings:**

In this nonrandomized controlled trial using national data from 1183 hospitals, hospitals that received negative publicly reported feedback improved a statistically significant 8.44 percentage points more on their EHR medication safety performance in the subsequent year compared with hospitals that received positive feedback.

**Meaning:**

These findings suggest that publicly reported quality feedback may be an effective tool to encourage hospital EHR medication safety alerts consistent with current standards.

## Teaser

This nonrandomized controlled trial evaluated the association of publicly reported feedback for hospital medication safety with improvement in the subsequent year.

## Introduction

The US federal government has spent more $30 billion to digitize the health care system by adopting electronic health records (EHRs).^1,2^ Despite this investment, the promise of EHRs to dramatically improve quality has remained elusive.^3^ One mechanism by which EHRs were expected to improve quality was the implementation of computerized provider order entry (CPOE), paired with clinical decision support (CDS) tools. Computerization of drug ordering in particular is associated with reduced rates of adverse drug events, which remain a significant source of patient harm.^4,5,6^

CPOE allows physicians and other clinicians to write orders for patients electronically, rather than through verbal or written communication. CDS linked with CPOE then uses EHR data about the patient as well as medication reference databases to supplement clinician decision-making and prevent potential adverse drug events, such as ordering a drug that the patient has a documented allergy to or is likely to have a negative interaction with another drug the patient is using.^7^ CDS tools intervene at the point of care and alert clinicians to potential adverse drug events before they happen. However, performance outcomes have been mixed, and a significant amount of customization happens at the organization level, resulting in heterogeneity even within hospitals using the same technology.^6,7,8,9^ As a result, while medication-related safety performance has improved, there is major progress to be made, with hospitals correctly alerting clinicians to fewer than two-thirds of potential adverse drug events.^8^

One potential policy mechanism to incentivize quality improvement is public reporting of performance. Public quality reporting has been theorized to reduce information asymmetries and increase patient welfare while providing low-performing hospitals incentive to improve.^10,11,12,13^ However, recent evidence has cast doubt on whether national programs are useful tools in identifying high quality,^14^ and while some programs, such as the Joint Commission, do provide a quality floor that all nearly all hospitals must meet, they may not help distinguish higher or lower quality among accredited hospitals, and it is unclear if public reporting is an effective way to encourage improvement. Public quality reporting may also unfairly penalize hospitals that serve low-income and disadvantaged populations,^15,16^ and result in patient selection to avoid patients who are more seriously ill.^17^ While hospitals may improve when they participate in these programs,^18,19^ pay-for-performance initiatives intended to reward high quality through financial incentives have found mixed results,^20^ with the potential for hospitals to game these systems and harm patients.^21^

Little evidence exists regarding the impact of public EHR quality reporting. The largest national evaluation of EHR safety is the CPOE Evaluation Tool of the Leapfrog Hospital Survey, an assessment using simulated patients and orders to evaluate whether the hospital EHR correctly generates CDS alerts for potential adverse drug events.^22^ The evaluation is derived from historical patients and orders that caused patient harm, and the results of the CPOE evaluation are included as one of many quality measures publicly reported on Leapfrog’s website. Empirical evidence has found the Leapfrog ratings are associated with outcome quality,^23^ and that performance on the CPOE Evaluation Tool was correlated with lower rates of adverse drug events,^24^ with some evidence suggesting that hospitals who use the CPOE Evaluation Tool multiple times improve with experience.^6^ However, to our knowledge, there has been no examination of whether the CPOE Evaluation Tool encourages quality improvement.

We used national data from the Leapfrog CPOE Evaluation Tool to identify the association of publicly reported feedback with EHR medication safety performance. Leveraging a change in the scoring in the CPOE Evaluation in 2017, we used a regression discontinuity design to answer 2 research questions; first, do hospitals that receive negative feedback regarding their safety performance improve more in the subsequent year compared with hospitals that receive positive feedback, and second, do hospitals make those improvements in basic or advanced CDS capabilities?

## Methods

This nonrandomized controlled trial was deemed exempt from ethics board review by the University of Utah institutional review board, the institution that facilitated the data collection. The need for informed consent was waived per institutional policy because this study did not involve any real patients or real patient data. This study is reported following the Transparent Reporting of Evaluations with Nonrandomized Designs (TREND) reporting guideline.

### Design and Administration of the CPOE Evaluation Tool

The CPOE Evaluation Tool is a test designed by researchers at the Brigham and Women’s Hospital and University of Utah and administered by the Leapfrog Group.^22^ The CPOE Evaluation Tool is included as part of the Leapfrog Hospital Survey and is one of several process quality measures used by the Leapfrog Group in their evaluation and rating of US hospitals.

The CPOE Evaluation Tool uses simulated patients and medication orders, input into the hospital’s EHR system, that mimic the experience of a clinician writing orders for actual patients to evaluate safety performance. The simulated patients and orders were designed to test the performance of CPOE paired with CDS to prevent potential adverse drug events most likely to cause serious harm to patients, and the orders were based on real-world incidents of preventable adverse drug events from patients who experienced serious harm or death.^9,25^ Simulated orders were divided into subcategories based on the type of adverse drug events they represented and grouped into 2 categories: orders with potential adverse events prevented by basic CDS (ie, drug-allergy, drug-route, drug-drug, drug-dose for single doses, and therapeutic duplication) and those that require advanced CDS (ie, drug-laboratory, drug-dose for daily doses, drug-age, drug-diagnosis, and corollary orders contraindications).^25^ The number of orders per hospital varies, and if a medication used in an order is not on the hospitals formulary or otherwise not prescriptible, it is excluded. The primary outcome measure was whether the hospital EHR system correctly generated an alert, either a CDS pop-up alert or a hard stop that prevented the clinician from submitting the order after entering an order that could result in an adverse drug event. The overall score, expressed as a percentage, is the number of orders correctly alerted on divided by the number of total orders that should generate alerts.

Administration of the test is performed by a team of hospital representatives at each participating hospital, with a clinician entering test medication orders into the EHR and recording in detail how the EHR responds. All simulated patients and orders in the evaluation are input during a single session. A hospital representative enters those responses into the evaluation tool and is presented with a qualitative feedback score, as well as percentage scores across the subcategories, but not the details of individual orders the hospital EHR did not correctly alert on, except in the case of orders in which the error would likely result in a fatality. The qualitative feedback scores for the 2017 test were “Full Demonstration of Safety Standards” for hospitals whose overall score was 50% or greater, “Substantial Demonstration of Safety Standards” for hospitals between 30% and 49.99%, and “Some Demonstration of Safety Standards” for hospitals less than 30%. Prior to 2017, the overall score and qualitative feedback was generated based on a different formula without sharp cutoff points. To determine whether hospitals are overalerting on safe orders that should not generate alerts, the test also includes several nuisance orders that would not cause any patient harm. If hospitals alert on these orders, they appear in the hospital nuisance score but does not impact their overall score.^26^ To prevent gaming of the test, several control orders that should not generate CDS alerts are included, and the test process is timed so that hospitals cannot take longer than 6 hours to complete the full test and input all of the simulated orders, although most hospitals complete the test in 2 to 3 hours. Hospitals that exceed the time threshold or report alerts on too many control orders are disqualified, although this amounts to less than 1% of hospitals each year. The Leapfrog Group audits hospitals to ensure accuracy.

### Data Collection and Sample

The sample included all hospitals that took the Leapfrog CPOE Evaluation Tool in both calendar year 2017 and 2018. These data were merged with American Hospital Association Annual Survey data from 2017 and 2018 to capture hospital demographic information. The final analytic sample included 1183 hospitals in a balanced panel from January 1, 2017 to December 31, 2018.

### Statistical Analysis

We calculated descriptive statistics for hospitals in the sample taking the Leapfrog CPOE Evaluation Tool in calendar years 2017 and 2018, including mean overall score and SD in both years as well as mean change in score between years. We also described the sample by hospital characteristics, including size, teaching status, health system membership, rural vs urban location, and region in the United States.

To estimate the association of providing hospitals with qualitative feedback on safety performance, we used a sharp regression discontinuity design using the 50% cutoff for Full Demonstration feedback. This compares hospitals that narrowly received the negative Substantial Demonstration feedback with hospitals that narrowly received the Full Demonstration feedback. The identifying assumption was that hospitals on either side of the cutoff were similar across other measures that might impact performance.^27,28,29^

We estimated the standard ordinary least squares regression discontinuity model,^30^ in which the dependent variable was overall CPOE Evaluation Tool score change from 2017 to 2018, expressed as percentage points. Our independent variable of interest was a binary indicator for whether the hospital received the Substantial Demonstration feedback, and the running variable was overall CPOE Evaluation score in 2017. We estimated this model with and without hospital demographic characteristics as controls. We then estimated the local average treatment effect nonparametrically using local polynomial inference developed by Calonico et al.^31,32,33,34,35^ This model fits local linear regressions on either side of the cutoff, using a data-driven bandwidth selection procedure to optimize the bias-variance tradeoff. We estimated this model with and without hospital covariates (including hospital size, teaching status, health system membership, rurality, and census region) and with bias-adjusted robust SEs clustered at the hospital-level.

We then examined the mechanism by which improvement occurs using this model separately on basic and advanced CDS. We calculated separate scores, ranging from 0% to 100% in the same way as the overall score, separately for each component: the orders that fall under the basic CDS category, and then the orders in the advanced CDS category. All analysis was conducted in Stata statistical software version 16.1 (StataCorp) with the rdrobust package, with 2-sided α = .05. Data were analyzed from January through September 2020.

To ensure these results were robust to a wide array of different possible specifications, we estimated the model 60 different times, varying different aspects of the specification including bandwidth selection (using several manual as well as different data-driven algorithms), local polynomial order (local linear compared to quadratic),^36^ inclusion of covariates, kernel choice for weighting observations near the cutoff point (triangle, Epanechnikov, and uniform), and SE calculations. We then plotted the point estimates and 95% CIs for the treatment effect in a specification curve (eFigure 1 in the Supplement).

We conducted a series of tests to ensure that the assumptions necessary for association identification were likely to be met. First, to ensure that no manipulation of the running variable was present, we plotted the density of the overall score in 2017 and evaluated whether empirical evidence of manipulation was present using the procedure outlined by Cattaneo et al,^37^ building on the McCrary test,^38^ and found no evidence of manipulation (eFigure 2 in the Supplement). We plotted the distribution of the hospital demographics across the running variable (eFigure 3 in the Supplement). We conducted a series of placebo tests estimating the model at alternative cutoff points and found no statistically significant results at any of placebo cutoffs (eTable 1 in the Supplement). We ran several versions of the model testing EHR vendor effects, including subsamples using only the 3 most commonly used vendors (eTable 2 in the Supplement) as well as including dummy variables to control for vendor (eTable 3 in the Supplement). We also ran the model including previous experience with the Leapfrog CPOE Evaluation (eTable 4 in the Supplement). Finally, to test whether hospital improvement was associated with increased unnecessary nuisance alerts, we used the same sharp regression discontinuity model at the 50% treatment assignment cutoff on a dependent variable of nuisance alert score change and found no evidence this occurred (eFigure 4 in the Supplement).

## Results

### Sample Descriptive Statistics

A total of 1183 hospitals were included, with a mean (SD) 2017 COPE score of 59.3% (16.3%). Hospital overall scores on the CPOE Evaluation Tool improved to a mean (SD) of 66.5% (14.9%) in 2018. Most hospitals in the sample were medium-sized (ie, 100-399 beds; 721 hospitals [60.9%]), followed by large hospitals with more than 400 beds (247 hospitals [20.9%]) and then small hospitals with fewer than 100 beds (215 hospitals [18.2%]). Most hospitals in the sample were teaching hospitals (674 hospitals [57.0%]), members of a health system (187 hospitals [84.2%]), and located in urban areas (1047 hospitals [88.5%]) (Table 1).

**Table 1.** Sample Characteristics

Characteristic	Hospitals, No. (%) (N = 1183)
Leapfrog CPOE inpatient drug performance, mean (SD)	
Overall score	
2017	59.3 (16.3)
2018	66.5 (14.9)
Change in score, 2017-2018	7.24 (14.5)
Hospital size	
Small (<100 beds)	215 (18.2)
Medium (100-399 beds)	721 (60.9)
Large (≥400 beds)	247 (20.9)
Teaching status	
Nonteaching	509 (43.0)
Teaching	674 (57.0)
Health system membership	
No	187 (15.8)
Yes	996 (84.2)
Location	
Rural	136 (11.5)
Urban	1047 (88.5)
Region	
Northeast	236 (19.9)
West	308 (26.0)
Midwest	216 (18.3)
South	423 (35.8)

Abbreviation: CPOE, Computerized Provider Order Entry.

### Hospital Improvement in Response to Feedback

We identified a clear discontinuity in hospital improvement in the subsequent year at the cutoff point between Full Demonstration and Substantial Demonstration feedback in 2017 (Figure).^39^ The ordinary least squares model found a significant association of providing hospitals with the negative Substantial Demonstration feedback with improvement in the subsequent year without covariates (β = 4.71 [95% CI, 1.53 to 7.89]) and with covariates (β = 4.82 [95% CI, 1.65 to 8.01]). The Calonico et al^31,32,33,34,35^ model also found that hospitals that received the Substantial Demonstration feedback improved 8.44 (95% CI, 0.09-16.80) percentage points more compared with hospitals on the other side of the discontinuity (9.19 [95% CI, 0.36-18.02] percentage points with covariates) (Table 2).

**Figure.**
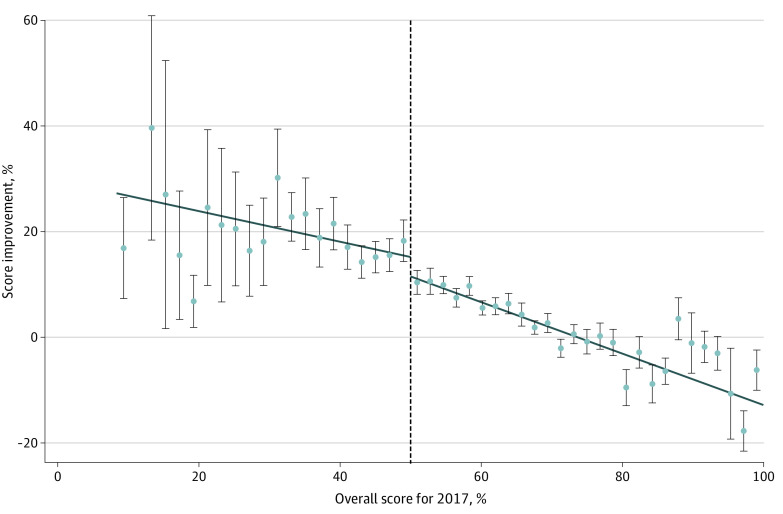
Hospital 2017 CPOE Performance Score and Improvement in 2018 Dots indicate mean scores; whiskers, 95% CIs; vertical line, cutoff point of 50% between hospitals that received the Full Demonstration and Substantial Demonstration feedback in 2017.

**Table 2.** Impact of Feedback on Hospital Performance Improvement

Model	β (95% CI), percentage points	*P* value
OLS	4.71 (1.53-7.89)	<.001
Nonparametric	8.44 (0.09-16.80)	.04
OLS (with covariates)	4.83 (1.65-8.01)	<.001
Nonparametric (with covariates)	9.19 (0.36-18.02)	.04

Abbreviation: OLS, ordinary least squares.

These results were robust to a wide array of modeling choices, shown in the specification curve in eFigure 1 in the Supplement. Of 60 different specifications tested, all but 3 produced a statistically significant result, and the 3 tests that were not significant were directionally consistent with other specifications. The results were also consistent when evaluating subsamples of the 3 largest EHR vendors (eTable 2 in the Supplement), as well as including controls for EHR vendor (eTable 3 in the Supplement) and previous Leapfrog CPOE Evaluation experience (eTable 4 in the Supplement).

### Improvement Mechanism

The primary mechanism for the observed change was through improvement in the safety performance of alerting on potential adverse drug events classified as basic CDS. Using the same robust, bias-corrected estimator with data-driven bandwidth selection, we observed a statistically significant change associated with receiving negative Substantial Demonstration feedback on hospital improvement in basic CDS performance both without covariates (β = 8.71 [95% CI, 1.67 to 18.73]) and with covariates (β = 8.80 [95% CI, 2.14 to 15.44]). There was no association of improvement with advanced CDS (without covariates: β = 6.15 [95% CI, −9.11 to 26.83]; with covariates: β = 8.73 [95% CI, −8.23 to 31.31]) (Table 3).

**Table 3.** Improvement Differences Across “Basic” and “Advanced” Decision Support Categories

Model	Basic decision support		Advanced decision support	
	β (95% CI)	*P* value	β (95% CI)	*P* value
Nonparametric	8.71 (1.67 to 18.73)	.03	6.15 (−9.11 to 26.83)	.42
Nonparametric (with covariates)	8.80 (2.14 to 15.44)	.01	8.73 (−8.23 to 31.31)	.51

## Discussion

The US health care system has made an enormous investment in EHRs, but it is not clear this has resulted in significant safety improvements. One policy lever to encourage quality improvement is public reporting, yet few studies have empirically examined the effectiveness of EHR-focused quality reporting, and most existing studies are descriptive reports of the state CPOE quality. Using a nonrandomized controlled regression discontinuity design, we evaluated the association of providing hospitals that participated in a voluntary EHR safety performance assessment with negative publicly reported feedback with quality improvement. We found that hospitals that received the negative feedback Substantial Demonstration of Safety Standards, rather than the positive Full Demonstration of Safety Standards, improved significantly more in the subsequent year. That improvement was driven by safety gains from basic CDS, such as drug-drug or drug-allergy contraindications, rather than advanced CDS, such as corollary orders or daily drug dosing contraindications.

These results contribute to our understanding of hospital quality measurement and publicly reported performance feedback, on which the literature on improvement is mixed. Many studies of hospital response to quality measurement have focused on pay-for-performance initiatives, such as the Hospital Readmission Reduction Program, that target outcome quality. Despite efforts to risk-adjust these programs, outcome quality is only partially within the control of hospitals, and measurement may be noisy.^40^ The result has been an ongoing debate over whether improvement in these programs could be a result of hospitals selecting against patients who are more seriously ill or otherwise gaming the measures rather than improving quality.^21,41,42^ In contrast, the Leapfrog CPOE Evaluation Tool is a measure of process quality that evaluates whether or not an alert is correctly triggered when best practices suggest an order may cause an adverse drug event. While process quality evaluations do not directly measure patient harm, they do accurately measure an aspect of care almost entirely within the control of the hospital.^43^ Therefore, hospitals are more likely to be able to respond to feedback and improve, rather than having incentive to simply select patients less likely to negatively impact their scores.

The Leapfrog CPOE Evaluation Tool differs from other quality evaluations in its scope, focusing narrowly on evaluating EHR performance in alerting clinicians to potential adverse drug events. This is in contrast to broad quality programs, like CMS Hospital Compare or the Joint Commission, which are composite measures of many aspects of quality. By providing feedback on a more targeted dimension of quality, hospitals may be more able to quickly identify opportunities for improvement and then act on those to improve their score in the next year. Hospitals that received negative feedback may have allocated more organizational resources on improving EHR medication safety or enabled stricter CDS alerting, thereby realizing nearly immediate quality gains. Our results showing that the mechanism for improved performance was basic decision support, which may be easier to build or enable within a single year, may support this hypothesis. Therefore, policy makers designing quality incentives may wish to consider targeted measures of process quality, like the Leapfrog CPOE Evaluation Tool. Additionally, future research should also examine whether improvements in process quality measures, such as the Leapfrog CPOE Evaluation, translate into improvements in outcome quality measures, such as adverse drug event rates, mortality, and patient experience.

### Limitations

This study has several limitations. First, regression discontinuity models estimate a local average treatment effect, and hospitals in different parts of the quality distribution may have different responses to feedback. Because so few hospitals scored less than 35% in 2017, we were unable to evaluate the association of receiving lower scores with subsequent quality improvement. Second, while the Leapfrog CPOE Evaluation Tool has been associated with actual reductions in preventable adverse drug events,^24^ it is a measure of process quality rather than outcome quality, and higher scores on the evaluation may not necessarily translate into better outcomes, and the most recent study to assess the association between Leapfrog CPOE Evaluation score and rates of adverse drug events in hospitals was published in 2013.^24^ Additionally, the Leapfrog CPOE Evaluation Tool does not capture forms of CDS that happen upstream of the order, such as the use of condition-specific order sets with appropriate defaults. Third, the Leapfrog CPOE Evaluation Tool is voluntary, and it is likely there is a selection into the sample in that hospitals that choose to participate in quality measurement are more motivated and likely to respond to negative feedback and improve. Fourth, while our analysis of nuisance alert scoring suggests that hospitals are not improving by burdening clinicians with low-value alerts, it is important to balance safety features and the potential impact on clinician well-being through alert fatigue and burnout.^44^ Fifth, because our identification takes advantage of a sharp cutoff in scoring that was new in 2017, we are only able to use 2 years of data rather than the full history of Leapfrog CPOE evaluations.^45,46^

## Conclusions

This nonrandomized controlled trial using data from a national evaluation of EHR medication safety found that hospitals that received publicly reported negative feedback improved quality 8.4 percentage points more than those that received positive feedback in the subsequent year. This outcome was driven by improvement in basic CDS, rather than more advanced CDS capabilities. Despite this progress, there is still considerable room for improvement, with few hospitals receiving a perfect score, suggesting that all hospitals may benefit from continued assessment of this type. These results suggest that publicly reported feedback on specific dimensions of quality may lead to improvement.

## Notes

Supplement. **eFigure 1.** Specification Curve of Main Result Robustness **eFigure 2.** Density Plot and McCrary Test **eFigure 3.** Covariates Across the Cutoff Point **eTable 1.** Regression Discontinuity Model Placebo Test **eTable 2.** Subsamples by EHR Vendor **eTable 3.** Main Results with EHR Vendor Controls **eTable 4.** Main Results with Leapfrog CPOE Evaluation Experience Controls **eFigure 4.** Alert Fatigue Mechanism Analysis
