# Supplementary material for: Association of Hospital Public Quality Reporting With Electronic Health Record Medication Safety Performance
Source: JAMA Netw Open. 2021 Sep 21;4(9):e2125173. doi: 10.1001/jamanetworkopen.2021.25173 (PMC8456388; doi:10.1001/jamanetworkopen.2021.25173)
Supplement: Supplement. — eFigure 1. Specification Curve of Main Result Robustness eFigure 2. Density Plot and McCrary Test eFigure 3. Covariates Across the Cutoff Point eTable 1. Regression Discontinuity Model Placebo Test eTable 2. Subsamples by EHR Vendor eTable 3. Main Results with EHR Vendor Controls eTable 4. Main Results with Leapfrog CPOE Evaluation Experience Controls eFigure 4. Alert Fatigue Mechanism Analysis [file jamanetwopen-e2125173-s001.pdf]

Supplemental Online Content

Holmgren AJ, Bates DW. Association of hospital public quality reporting with electronic health record medication safety performance. *JAMA Netw Open*. 2021;4(9):e2125173. doi:10.1001/jamanetworkopen.2021.25173

eFigure 1. Specification Curve of Main Result Robustness
eFigure 2. Density Plot and McCrary Test
eFigure 3. Covariates Across the Cutoff Point
eTable 1. Regression Discontinuity Model Placebo Test
eTable 2. Subsamples by EHR Vendor
eTable 3. Main Results with EHR Vendor Controls
eTable 4. Main Results with Leapfrog CPOE Evaluation Experience Controls
eFigure 4. Alert Fatigue Mechanism Analysis

This supplemental material has been provided by the authors to give readers additional information about their work.

© 2021 Holmgren AJ et al. *JAMA Network Open.*

eFigure 1. Specification Curve of Main Result Robustness

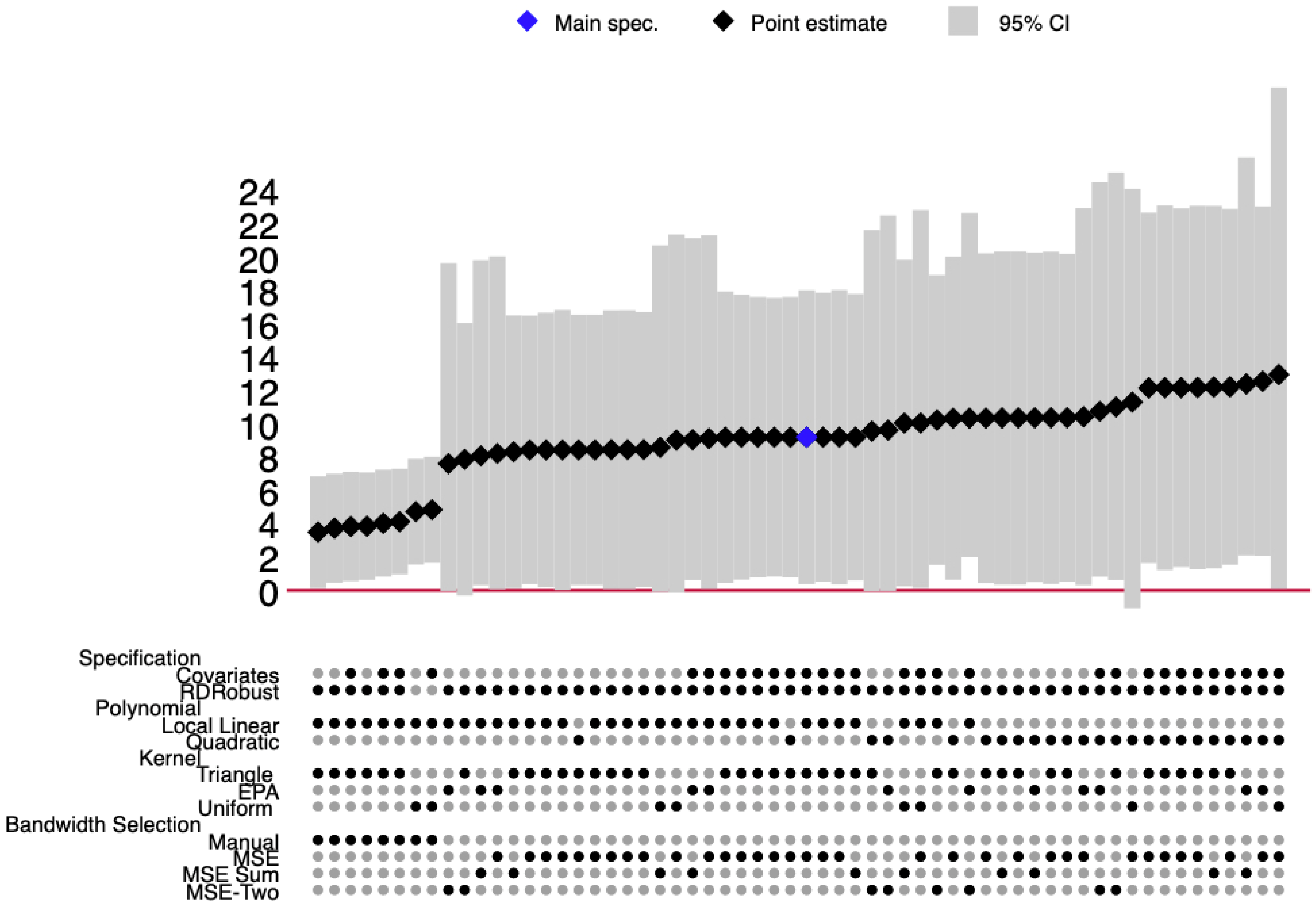

© 2021 Holmgren AJ et al. *JAMA Network Open.*

Caption for Appendix Exhibit 1: This specification curve plots the point estimate and 95% confidence interval for 60 different potential specifications of the primary model of interest, with the dependent variable of hospital improvement in Leapfrog CPOE Evaluation Tool score from 2017 to 2018 and the treatment being the discontinuity between the top score ("Full Demonstration of Safety Standards") and the score below 50%, "Substantial Demonstration of Safety Standards." The bottom table shows the features of each model, fully described below:

Specification
Covariates: indicates whether the model included covariates as controls. Covariates are listed in Table 1, and include hospital size, teaching status, health system membership, rural vs urban location, and census region in the United States.

RDRobust: indicates whether the model was estimated with the non-parametric method developed by Calonico et al., implemented via the RDRobust package in Stata. If this indicator is not shaded, the standard ordinary least squares regression discontinuity model was used.

Polynomial
For the non-parametric Calonico et al RDRobust model, specifies what order of local polynomial was fit at the discontinuity.

Kernel
Specifies what kernel weighting method was used for weighting observations near the cut-off point of the discontinuity. Triangle is the default and weights observations closer to the discontinuity more heavily. Uniform kernel indicates all observations being given equal weight. EPA stands for the Epanechnikov kernel (see more at Samiuddin, M., & El-Sayyad, G. M. (1990). On nonparametric kernel density estimates. Biometrika, 77(4), 865-874.)

Bandwidth Selection
Specifies what method was used to select the bandwidth around the discontinuity cut-off point for fitting the local linear polynomial.

Manual: Manually input bandwidth.

MSE: Mean-squared error (MSE) bandwidth computation optimizes the bias-variance trade-off by selecting the MSE-optimal bandwidth for treatment effect identification. This is the default for the RDRobust package.

MSE Sum: Similar to the MSE-optimal bandwidth computation, but selects for the sum of the regression estimates rather than the difference. '

MSE-Two: Similar to the MSE-optimal bandwidth computation, but calculates MSE-optimal bandwidth selectors separately above and below the cut-off.

For more information, see Calonico S, Cattaneo MD, Titiunik R. Robust Nonparametric Confidence Intervals for Regression-Discontinuity Designs: Robust Nonparametric Confidence Intervals. Econometrica. 2014;82(6):2295-2326. doi:10.3982/ECTA11757

© 2021 Holmgren AJ et al. *JAMA Network Open.*

eFigure 2. Density Plot and McCrary Test

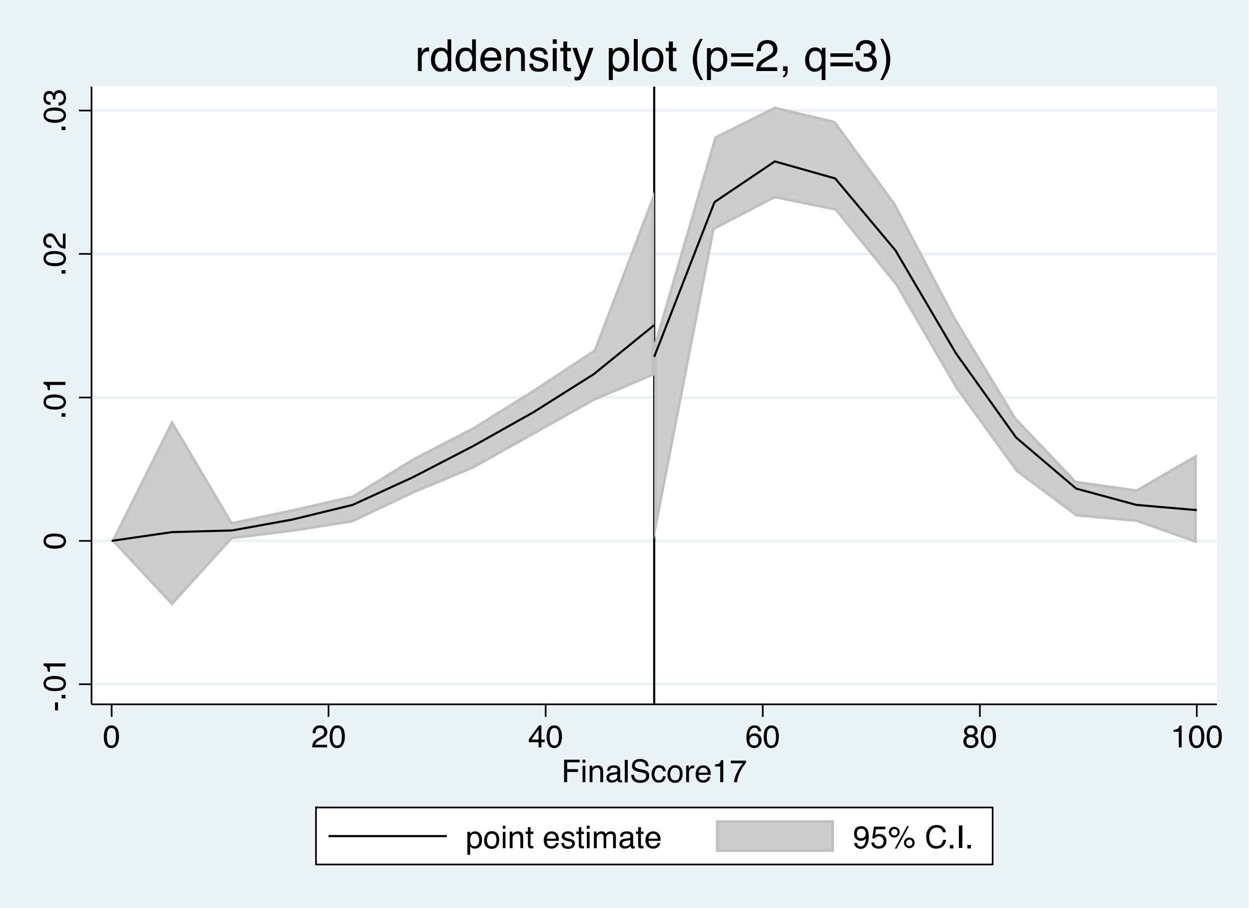

T Statistic: 0.3340
P-value: 0.7384

Do not reject the null: no statistical evidence of systematic manipulation of the running variable.

© 2021 Holmgren AJ et al. *JAMA Network Open.*

eFigure 3. Covariates Across the Cutoff Point

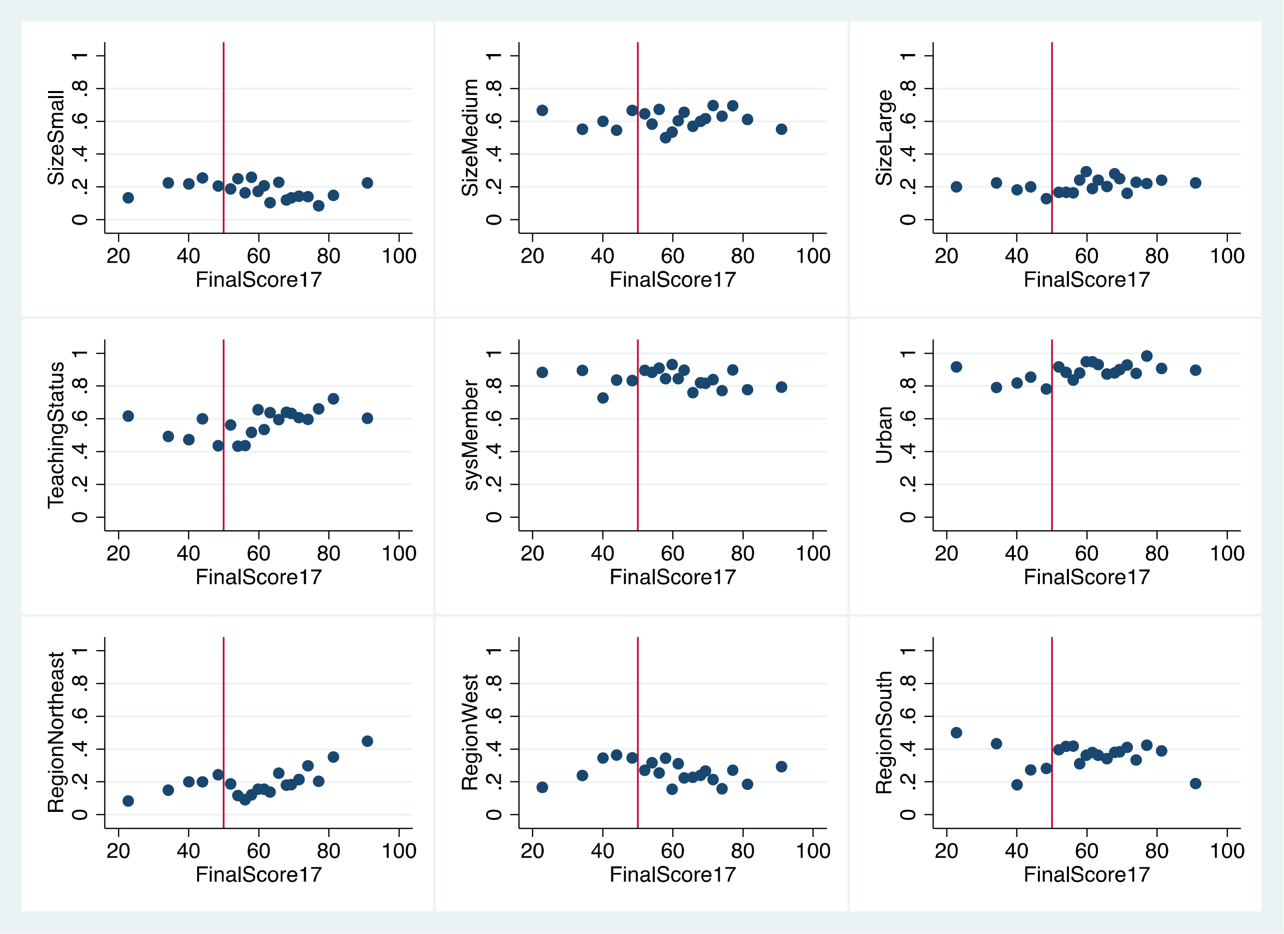

RDD estimates using hospital demographic covariates as dependent variables with the cut-off between "Full Demonstration" and "Substantial Demonstration". All estimates use the Calonico et al rdrobust estimator with bias-corrected standard errors.

**Hospital Size**	Coefficient	P-Value
Small Hospitals (<100 Beds)	-0.03	0.87
Medium Hospitals (100 - 399 Beds)	-0.16	0.19
Large Hospitals (400+ Beds)	0.23	0.10
**Teaching Status**		
Teaching Hospital	0.07	0.18
**Health System Membership**		
Health System Member	0.08	0.52
**Location**		
Urban	0.62	0.06
**Region**		

© 2021 Holmgren AJ et al. *JAMA Network Open.*

Northeast	-0.15	0.20
West	-0.03	0.94
Midwest	-0.012	0.90
South	0.195	0.20

No significant differences that would lead to bias in our main result.

© 2021 Holmgren AJ et al. *JAMA Network Open.*

eTable 1. Regression Discontinuity Model Placebo Test

	Coef.	p-value	[95% Conf.	Interval]
**Placebo Cutpoints**				
Cutoff: 25%	25.23	0.12	-8.28	75.75
Cutoff: 45%	5.80	0.10	-1.33	14.81
Cutoff: 65%	-1.04	0.54	-5.03	2.62
Cutoff: 90%	-23.90	0.45	-116.96	51.33

All models use the rdrobust data-driven bandwidth estimator with robust standard errors clustered at the hospital level.

© 2021 Holmgren AJ et al. *JAMA Network Open.*

eTable 2. Subsamples by EHR Vendor

	Performance Improvement				
	Vendor A Only	Vendor B Only	Vendor C Only	Vendors A, B, and C Only	All Other Vendors Only
**Negative Feedback**	13.56	8.67*	16.00	10.11**	8.50
**95% Confidence Interval**	(-19.52 - 49.89)	(-1.07 - 18.41)	(-5.81 - 37.81)	(1.06 - 23.68)	(-5.93 - 22.93)
**Observations**	1183	1183	1183	1183	1183
**Effective Observations**	39	149	44	204	66
**Controls**	No	No	No	No	No
**Robust Bias Corrected Bandwidth Selection**	Yes	Yes	Yes	Yes	Yes

w/ covariates	Performance Improvement				
	Vendor A Only	Vendor B Only	Vendor C Only	Vendors A, B, and C Only	All Other Vendors Only
**Negative Feedback**	33.88*	8.22*	21.68**	11.75**	7.27
**95% Confidence Interval**	(-5.52 - 73.28)	(-0.89 - 17.33)	(1.81 - 41.54)	(1.61 - 26.34)	(-5.53 - 20.07)
**Observations**	1183	1183	1183	1183	1183
**Effective Observations**	31	145	39	190	55
**Controls**	Yes	Yes	Yes	Yes	Yes
**Robust Bias Corrected Bandwidth Selection**	Yes	Yes	Yes	Yes	Yes

*** $p < 0.01$ ** $p < 0.05$ * $p < 0.10$

Vendor A is the most commonly represented EHR vendor in our sample, Vendor B is the second most common, and Vendor C is the third most common. Vendor identities are blinded due to our confidentiality agreement with the data provider.

© 2021 Holmgren AJ et al. *JAMA Network Open.*

eTable 3. Main Results with EHR Vendor Controls

Full Sample with EHR Vendor Dummy Controls		
	Model 1	*Model 2*
**Negative Feedback**	9.31**	9.44**
**95% Confidence Interval**	(0.53 - 21.35)	(0.68 - 18.21)
**Observations**	1183	1183
**Effective Observations**	238	231
**Demographic Controls**	No	Yes
**Robust Bias Corrected Bandwidth Selection**	Yes	Yes

*** $p < 0.01$ ** $p < 0.05$ * $p < 0.10$

© 2021 Holmgren AJ et al. *JAMA Network Open.*

eTable 4. Main Results with Leapfrog CPOE Evaluation Experience Controls

**Negative Feedback**	9.19**
**95% Confidence Interval**	(0.36 – 18.02)
**Observations**	1183
**Effective Observations**	231
**Demographic Controls**	Yes
**Robust Bias Corrected Bandwidth Selection**	Yes

Model includes indicator variables for number of years of experience with the Leapfrog CPOE Evaluation up to and including 2018 as control variables.

*** $p < 0.01$ ** $p < 0.05$ * $p < 0.10$

© 2021 Holmgren AJ et al. *JAMA Network Open.*

eFigure 4. Alert Fatigue Mechanism Analysis

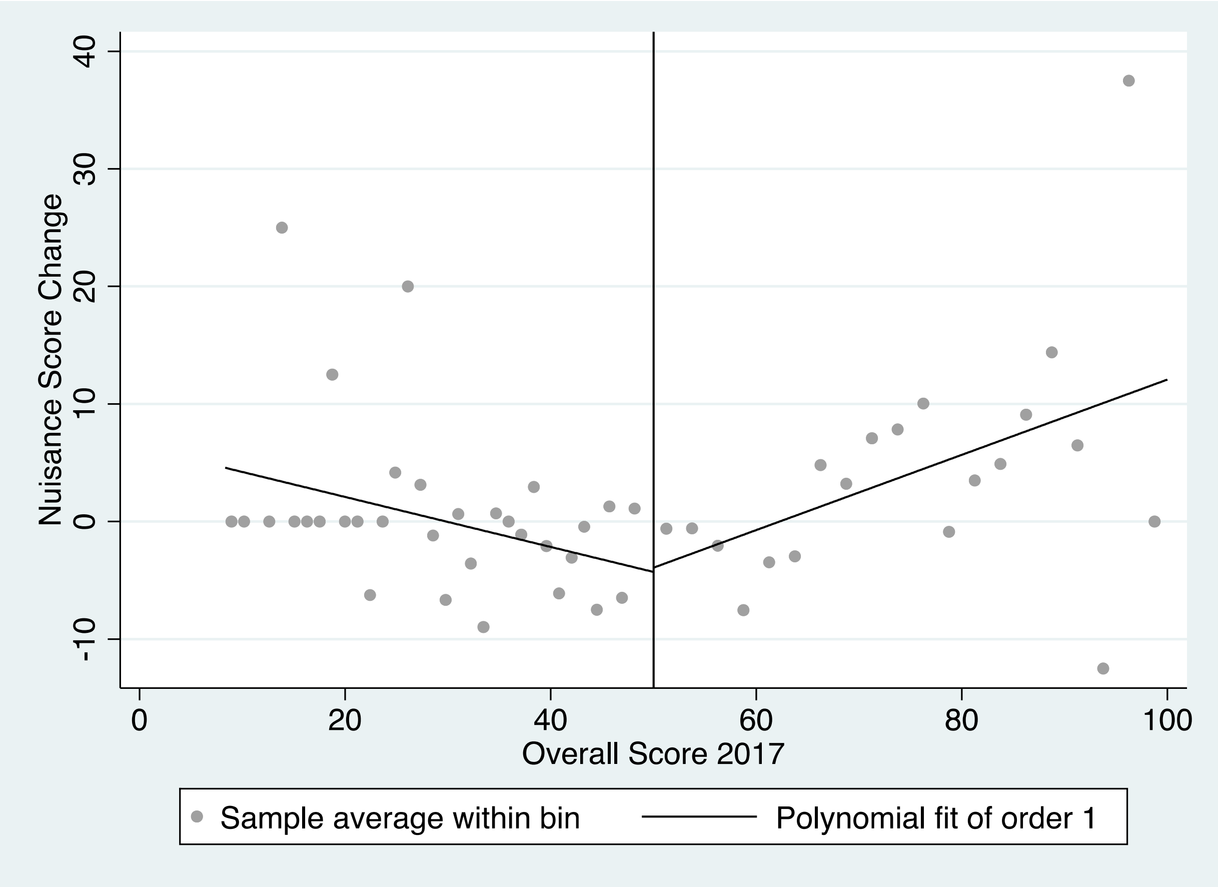

No significant effect in non-parametric RD model estimates.

© 2021 Holmgren AJ et al. *JAMA Network Open.*
